# Suppression of the hypothalamic-pituitary-thyroid axis is associated with the severity of prognosis in hospitalized patients with COVID-19

**DOI:** 10.1186/s12902-021-00896-2

**Published:** 2021-11-15

**Authors:** Juan Zheng, Zhenhai Cui, Ningjie Shi, Shenghua Tian, Ting Chen, Xueyu Zhong, Kangli Qiu, Jiaoyue Zhang, Tianshu Zeng, Lulu Chen, Huiqing Li

**Affiliations:** 1 Department of Endocrinology, Union Hospital, Tongji Medical College, Huazhong University of Science and Technology, Wuhan, 430022 China; 2 Hubei provincial Clinical Research Center for Diabetes and Metabolic Disorders, Wuhan, China

**Keywords:** Coronavirus disease 2019, Hypothalamic-pituitary-thyroid axis, Non-thyroidal illness syndrome, Subclinical hypothyroidism

## Abstract

**Background:**

The outbreak of severe acute respiratory syndrome novel coronavirus 2 (SARS-CoV-2) has spread rapidly worldwide. SARS-CoV-2 has been found to cause multiple organ damage; however, little attention has been paid to the damage to the endocrine system caused by this virus, and the subsequent impact on prognosis. This may be the first research on the hypothalamic-pituitary-thyroid (HPT) axis and prognosis in coronavirus disease 2019 (COVID-19).

**Methods:**

In this retrospective observational study, 235 patients were admitted to the hospital with laboratory-confirmed SARS-CoV-2 infection from 22 January to 17 March 2020. Clinical characteristics, laboratory findings, and treatments were obtained from electronic medical records with standard data collection forms and compared among patients with different thyroid function status.

**Results:**

Among 235 patients, 17 (7.23%) had subclinical hypothyroidism, 11 (4.68%) severe non-thyroidal illness syndrome (NTIS), and 23 (9.79%) mild to moderate NTIS. Composite endpoint events of each group, including mortality, admission to the ICU, and using IMV were observed. Compared with normal thyroid function, the hazard ratios (HRs) of composite endpoint events for mild to moderate NTIS, severe NTIS, subclinical hypothyroidism were 27.3 (95% confidence interval [CI] 7.07–105.7), 23.1 (95% CI 5.75–92.8), and 4.04 (95% CI 0.69–23.8) respectively. The multivariate-adjusted HRs for acute cardiac injury among patients with NTF, subclinical hypothyroidism, severe NTIS, and mild to moderate NTIS were 1.00, 1.68 (95% CI 0.56–5.05), 4.68 (95% CI 1.76–12.4), and 2.63 (95% CI 1.09–6.36) respectively.

**Conclusions:**

Our study shows that the suppression of the HPT axis could be a common complication in COVID-19 patients and an indicator of the severity of prognosis. Among the three different types of thyroid dysfunction with COVID-19, mild to moderate NTIS and severe NTIS have a higher risk of severe outcomes compared with subclinical hypothyroidism.

## Background

Coronavirus disease 2019 (COVID-19), a new global pandemic that first emerged in December 2019, is caused by severe acute respiratory syndrome coronavirus 2 (SARS-CoV-2). Due to its abrupt outbreak, rapid spread, and striking mortality, COVID-19 has drawn wide attention from various countries and regions, with more than 224 million confirmed cases and more than 4.6 million deaths as of September 12, 2021 [1]. One of the reasons for this catastrophic outcome is that SARS-CoV-2 attacks the body’s major organs through angiotensin-converting enzyme 2 (ACE2), a main viral entry receptor that is widely distributed in the lungs, heart, liver, and kidney, and causes damage to multiple organs [2]. However, little attention has been paid to the damage to the endocrine system caused by this virus, and the subsequent impact on prognosis.

In a previous study on endocrine dysfunction caused by severe acute respiratory syndrome (SARS) [3], the hypothalamic-pituitary-adrenal (HPA) and hypothalamic-pituitary-thyroid (HPT) axis were candidate targets for SARS-CoV. This study observing 61 SARS survivors found that 6.7% of patients had hypothyroidism including central hypothyroidism and primary hypothyroidism. Severe airway obstruction, hypoxemia, systemic inflammation, and glucocorticoid use may easily develop into subclinical hypothyroidism, overt hypothyroidism, and non-thyroid illness syndrome [4]. Non-thyroid illness syndrome (NTIS) was defined as the decreased concentrations of plasma tri-iodothyronine, low thyroxine, and normal range or slightly decreased concentration of thyroid-stimulating hormone [5]. Non-thyroid illness syndrome (NTIS) is common in patients with chronic obstructive pulmonary disease (COPD) regardless of whether the disease is stable or aggravated [6]. Several systemic inflammatory cytokines, such as IL-6, IL-1 and TNF-α, which are observed at elevated levels among patients with COPD, inhibit TSH synthesis or secretion [4, 6]. SARS-CoV-2 could induce reversible thyroid dysfunction, and the low T3 syndrome could be associated with the severity of COVID-19 [7]. These studies have shown that the HPT axis is altered in patients with SARS or COPD. In clinical experiences, we found that some patients with COVID-19 had suppressed thyroid function, suggesting that the HPT axis may be affected by SARS-CoV-2. However, little is known about the relationship between COVID-19 and the HPT axis.

In the present study, according to the results of thyroid function tests, patients were divided into four categories: normal thyroid function (NTF), subclinical hypothyroidism, mild to moderate NTIS, and severe NTIS. We then compared the clinical characteristics, laboratory findings, medication use, complications, and composite endpoint events of each group. Therefore, the aim of this study is to evaluate whether changes in the HPT axis are associated with the severity of prognosis of COVID-19 and to explore the risk factors associated with these changes.

## Methods

### Study design and participants

This is a retrospective study conducted in the West Campus unit of Wuhan Union Hospital. A total of 300 COVID-19 inpatients were admitted and received treatment between January 25, 2020 and March 17, 2020. These patients were diagnosed for COVID-19 based on the detection SARS-CoV-2, following the interim guidelines outlined by the World Health Organization (WHO) [8]. Prior to the enrollment of these inpatients in the study, they were subjected to an investigation of their thyroid function. Off these, a total of 235 patients were included in the analysis and remaining 65 inpatients were excluded from the study for either of the following reasons: (i) 13 patients had incomplete medical history/treatment history, (ii) 16 patients had pre-existing thyroid dysfunction, (iii) 5 patients had hyperthyroidism, (iv) 13 female patients were found to be pregnant, (v) 7 patients had end-stage of chronic organ dysfunction, (vi) the 11 patients were administered dopamine treatment before thyroid function test.

The current study was reviewed and approved by the ethics committee of Tongji Medical College, Huazhong University of Science and Technology, Wuhan, P. R China. Written informed consent was waived by the ethics committee of the designated hospital under the criteria of emerging infectious diseases. The clinical data of enrolled participants were anonymously treated and analyzed. As of April 4, 2020, among 235 inpatients included in the study, 6.8% (16/235) died and 93.2% (219/235) recovered.

### Data collection

The data was collected by a standardized electronic medical record data collection form. A well-trained team of doctors and researchers from Wuhan Union Hospital independently input and cross-checked the data into a computer database. Two researchers (C.Z., T.S.) in our team independently reviewed the clinical data of all laboratory-confirmed patients infected with SARS-CoV-2.

Basic information (age, sex, and shared medical history) was collected for each patient (addendum). Within three days after admission to the hospital, laboratory blood parameters including standard blood count (absolute white blood cells and lymphocytes, hemoglobin concentration, platelet count, arterial blood gas analysis, supply, and oxygen partial pressure [PaO2]), blood biochemistry (including liver and kidney function, creatine kinase, fasting plasma glucose, HbA1c, lactate dehydrogenase and electrolytes), coagulation, procalcitonin, c-reactive protein (CRP), erythrocyte sedimentation rate (ESR), myocardial enzyme spectrum, and thyroid function (free triiodothyronine, free thyroxine, and thyroid-stimulating hormone) were measured. No enrolled patients received dopamine treatment before thyroid function test and received thyroid hormone replacement therapy in hospital. Other data included medical imaging, treatment regimens (antiviral and antimicrobial agents), systemic corticosteroids, immunoglobulin G, respiratory support (such as nasal tubes, mask, high-flow nasal intubation, noninvasive and invasive mechanical ventilation), and prognosis (discharge or death).

### Classification of thyroid function abnormality

Patients were classified into four categories based on the reference range of thyroid function index in our hospital after admission: normal thyroid function (NTF) group, subclinical hypothyroidism group (FT3 within 2.63–5.7 pmol/L and FT4 within 9–19.18 pmol/L and TSH > 4.94 μIU/ml) [9], severe NTIS group (FT3 < 2.63 pmol/L and TSH < 0.35 μIU/ml; FT4 < 9 pmol/L and FT3 < 2.63 pmol/L and TSH < 0.35 μIU/ml; FT4 < 9 pmol/L and TSH < 0.35 μIU/ml) [10], and mild to moderate NTIS group (FT3 < 2.63 pmol/L or FT4 < 9 pmol/L and TSH within 0.35–4.94 μIU/ml) [10].

### Study outcomes

The primary component endpoint events included the use of invasive mechanical ventilation (IMV), admission to the intensive care unit (ICU), or death events. The duration of follow-up time for each patient (person-days) was calculated from the first diagnosis date of COVID-19 to the index date of discharge, death of inpatients, or transfer to other hospitals. We also assessed the complications of COVID-19 in the analyses. The definitions of SARS-CoV-2-associated acute respiratory distress syndrome (ARDS) and shock referred to the interim guidance of the WHO for SARS-CoV-2 [8]. Acute cardiac injury was defined as elevated serum levels of cardiac biomarkers (such as CK-MB and hypersensitive cardiac troponin I) above the 99th percentile reference limit, or new abnormalities found in the electrocardiogram and echocardiography [11]. Acute kidney injury was diagnosed according to KDIGO clinical practice guidelines [12]. According to the CURB-65 guidelines, the severity of COVID-19 was divided into two levels: mild, severe [13]. Clotting disease was diagnosed if prothrombin time (PT) was prolonged over 3 s or activation of partial thromboplastin time (APTT) was prolonged over 5 s [14]. Hypoproteinemia was defined as blood albumin less than 30 g/L.

### Statistical analysis

Differences in demographics, history of diseases, laboratory measurements, treatment and clinical outcomes among patients with the four types of thyroid function status (NTF, subclinical hypothyroidism, severe NTIS, and mild to moderate NTIS) were assessed using Pearson’s chi-square or Fisher’s exact test for categorical variables and the General Linear Model for continuous variables after adjustments for age and sex. Cox proportional hazards regression was used to estimate hazard ratios (HRs) of the composite endpoint of ICU, IMV, or death, and acute cardiac injury among patients with different thyroid function status. Three models were used: Model 1 adjusted for age and sex; Model 2 adjusted for age, sex, smoking, systolic blood pressure, fasting glucose, using antihypertensive medications, and using glucose-lowering drugs (36 patients were treated with insulin injection); and Model 3 adjusted for total cholesterol and using lipid-lowering agents (19 patients were treated with lipid-lowering agents). All the statistical analyses were performed with SPSS statistics V.25.0 for Windows (IBM). A two-sided *p* < 0.05 was considered statistically significant.

### Patient and public involvement

This was a retrospective study, no patients were directly involved in the study design, the research questions setting, or the outcome measurement. No patients were asked to advice on the interpretation or recording of the results.

## Results

General characteristics of the study population at baseline are given in Table 1. The median age of patients was 61 (interquartile range 51–69) years. Patients who had subclinical hypothyroidism, severe NTIS, and mild to moderate NTIS were slightly older, and were more likely to have histories of stroke and chronic kidney disease as compared with those in the normal group.

**Table 1:** Baseline characteristics according to different thyroid function categories among patients with COVID-19

	NTF	Subclinical hypothyroidism	Severe NTIS	Mild to moderate NTIS	*P* values
No. of participants	184	17	11	23	
Age, years	57.4 (0.99)	63.0 (3.27)	65.4 (4.06)	72.2 (2.81)	< 0.001
Men, %	88 (47.8)	6 (35.3)	6 (54.5)	12 (52.2)	0.696
History of chronic diseases, %					
Diabetes	27 (14.7)	2 (11.8)	2 (18.2)	5 (21.7)	0.734
Hypertension	50 (27.2)	6 (35.3)	6 (54.5)	11 (47.8)	0.063
Coronary heart disease	15 (8.2)	1 (5.9)	2 (18.2)	4 (17.4)	0.247
Stroke	3 (1.60)	0 (0.00)	2 (18.2)	4 (17.4)	0.001
Chronic pulmonary disease	12 (6.5)	0 (0.0)	2 (18.2)	0 (0.0)	0.156
Chronic liver disease	4 (2.2)	0 (0.0)	0 (0.0)	0 (0.0)	1.000
Chronic kidney disease	1 (0.5)	0 (0.0)	0 (0.0)	3 (13.0)	0.011
Cancer	9 (4.9)	1 (5.9)	2 (18.2)	2 (8.7)	0.168
Onset of symptom to, days					
Hospital admission	19.9 (0.92)	19.4 (3.17)	15.1 (4.08)	13.9 (2.66)	0.154
Confirmation of COVID-19	11.6 (0.86)	13.5 (2.96)	7.88 (3.80)	8.92 (2.48)	0.495

Data are means (SE) and are adjusted for age and sex unless otherwise indicated percentages. Abbreviations: *COVID-19* coronavirus disease 2019, *NTF* Normal thyroid function, *NTIS* Non-thyroidal illness syndrome

The laboratory parameters among COVID-19 patients are presented in Table 2. Patients with severe NTIS and patients with mild to moderate NTIS had higher mean values of CRP, white blood cell count, ESR, procalcitonin, cardiac troponin I, CKMB mass, prothrombin time, fibrinogen, lactate dehydrogenase, creatinine, blood urea nitrogen and fasting glucose than those of patients with either NTF or subclinical hypothyroidism. Patients with mild to moderate NTIS and patients with severe NTIS had lower mean values of albumin, total cholesterol and low-density lipoprotein cholesterol than those of patients with either NTF or subclinical hypothyroidism.

**Table 2:** Laboratory measurements according to different thyroid function categories among patients with COVID-19

	NTF	Subclinical hypothyroidism	Severe NTIS	Mild to moderate NTIS	*P* values
No. of participants	184	17	11	23	
White blood cell count, ×10^9^/L	6.26 (0.20)	6.52 (0.66)	9.33 (0.81)	7.59 (0.59)	0.001
Neutrophil count, ×10^9^/L	4.23 (0.19)	4.41 (0.60)	8.14 (0.75)	6.22 (0.54)	< 0.001
Lymphocyte count, ×10^9^/L	1.50 (0.07)	1.48 (0.21)	0.71 (0.27)	0.88 (0.19)	0.002
CRP, mg/L	17.6 (2.59)	7.65 (8.25)	82.0 (10.3)	67.4 (7.37)	< 0.001
Prothrombin time, s	13.0 (0.30)	13.0 (0.99)	17.8 (1.16)	13.9 (0.90)	0.001
ESR, mm/h	43.9 (2.73)	31.0 (9.23)	55.3 (13.8)	81.5 (8.50)	< 0.001
APTT, s	39.7 (2.26)	35.1 (7.61)	39.8 (8.91)	44.3 (6.88)	0.843
D-Dimer > 0.5 mg/L, %	57 (38.0)	8 (72.7)	5 (62.5)	14 (77.8)	0.001
Fibrinogen, g	3.79 (0.08)	3.29 (0.27)	4.72 (0.32)	3.88 (0.25)	0.009
Thrombin time, s	16.4 (0.71)	15.4 (2.40)	16.5 (2.81)	16.3 (2.17)	0.982
ALT, U/L	43.1 (2.46)	33.4 (8.05)	46.7 (10.0)	45.8 (7.18)	0.631
AST, U/L	32.5 (2.32)	29.9 (7.59)	54.4 (9.43)	54.4 (6.77)	0.004
Blood urea nitrogen, mmol/L	5.70 (0.37)	7.46 (1.21)	11.3 (1.51)	7.62 (1.08)	0.002
Creatinine, μmol/L	70.0 (2.00)	66.2 (6.53)	107.2 (8.12)	81.6 (5.83)	< 0.001
Lactate dehydrogenase, U/L	226 (9.08)	216 (29.1)	485 (36.2)	387 (26.0)	< 0.001
Albumin, g/L	34.8 (0.44)	34.6 (1.44)	31.0 (1.79)	30.3 (1.28)	0.004
Total cholesterol, mmol/L	4.63 (0.09)	4.97 (0.29)	4.04 (0.33)	3.95 (0.24)	0.012
Triglyceride, mmol/L	1.72 (0.07)	1.62 (0.23)	1.58 (0.26)	1.53 (0.19)	0.802
HDL-C, mmol/L	1.11 (0.03)	1.12 (0.08)	0.94 (0.10)	0.93 (0.07)	0.050
LDL-C mmol/L	2.74 (0.07)	3.09 (0.24)	2.32 (0.27)	2.20 (0.20)	0.014
Cystatin C, mg/L	0.93 (0.13)	2.44 (0.42)	1.32 (0.52)	1.12 (0.37)	0.008
Fasting glucose, mmol/L	6.25 (0.19)	6.01 (0.61)	8.85 (0.76)	8.69 (0.54)	< 0.001
Procalcitonin, ng/ml	0.11 (0.16)	0.10 (0.55)	3.04 (0.69)	1.12 (0.49)	< 0.001
BNP, pg/ml	76.8 (26.7)	201.2 (64.2)	100.9 (61.3)	120.4 (54.2)	0.349
Cardiac Troponin I, ng/l	10.4 (25.7)	4.66 (77.0)	39.2 (98.4)	220.6 (66.0)	0.038
CKMB mass, ng/ml	0.85 (0.17)	1.81 (0.51)	2.85 (0.62)	2.55 (0.43)	< 0.001
CKMB activity, U/L	12.8 (0.84)	13.7 (2.73)	16.3 (3.54)	17.7 (2.53)	0.286
FT3, pmol/L	4.20 (0.05)	4.33 (0.16)	2.44 (0.20)	2.20 (0.14)	< 0.001
FT4, pmol/L	13.0 (0.13)	12.7 (0.42)	12.6 (0.52)	11.9 (0.37)	0.047
THS, μIU/ml	2.08 (0.08)	7.06 (0.27)	0.13 (0.33)	1.26 (0.24)	< 0.001

Data are means (SE) and are adjusted for age and sex unless otherwise indicated percentages. Abbreviations: *COVID-19* coronavirus disease 2019, *NTF* Normal thyroid function, *NTIS* Non-thyroidal illness syndrome, *CRP* C-reactive protein, *ESR* Erythrocyte sedimentation rate, *APTT* Activation of partial thromboplastin time, *ALT* Alanine aminotransferase, *AST*, Aspartate aminotransferase, *HDL-C* High-density lipoprotein cholesterol, *LDL-C* Low-density lipoprotein cholesterol, *BNP* B-type natriuretic peptide, *FT3* Free Triiodothyronine, *FT4* Free Thyroxine, *TSH* Thyroid-stimulating hormone

Table 3 shows the treatments and outcomes among COVID-19 patients with different thyroid function status. Patients with severe NTIS and mild to moderate NTIS had higher incidences of COVID-related complications, including ARDS (9.1–13.0% vs 0.0–1.1%), acute cardiac injury (54.5–70.0% vs 15.3–23.5%), acute kidney injury (21.7–27.3% vs 0.0–2.7%), shock (36.4–47.8% vs 0.0–1.6%), hypoalbuminemia (45.5–52.2% vs 18.6–23.5%), and coagulopathy (27.3–30.0% vs 0.0–10.9%), as well as higher severe types of COVID-19 (100% vs 75.5–76.5%) compared to patients with NTF or subclinical hypothyroidism. Patients with severe NTIS or mild to moderate NTIS were more likely to use antibiotic therapy, corticosteroid treatment, and intravenous immunoglobin treatment during hospital admission, compared to patients with NTF or subclinical hypothyroidism.

**Table 3:** Treatments and outcomes according to different thyroid function categories among patients with COVID-19

	NTF	Subclinical hypothyroidism	Severe NTIS	Mild to moderate NTIS	*P* values
No. of participants	184	17	11	23	
Treatments, %					
Antiviral therapy	162 (88.0)	15 (88.2)	8 (72.7)	20 (87.0)	0.455
Antibiotic therapy	104 (56.5)	8 (47.1)	9 (81.8)	20 (87.0)	0.010
Use of corticosteroid	37 (20.1)	4 (23.5)	6 (54.5)	12 (52.2)	0.001
Intravenous immunoglobin	18 (9.8)	3 (17.6)	5 (45.5)	11 (47.8)	< 0.001
Antihypertensive medicine	56 (30.4)	10 (58.8)	4 (36.4)	9 (39.1)	0.110
Glucose-lowering medicine	35 (19.0)	1 (5.9)	2 (18.2)	6 (26.1)	0.451
Lipid-lowering medicine	16 (8.7)	0 (0.0)	1 (9.1)	2 (8.7)	0.686
Illness severity, %					0.005
Non-severe	45 (24.5)	4 (23.5)	0 (0.0)	0 (0.0)	0.005
Severe	139 (75.5)	13 (76.5)	11 (100.0)	23 (100.0)	0.005
Complications, %					
ARDS	2 (1.1)	0 (0.0)	1 (9.1)	3 (13.0)	0.009
Acute cardiac injury	27 (15.3)	4 (23.5)	7 (70.0)	12 (54.5)	< 0.001
Acute kidney injury	5 (2.7)	0 (0.0)	3 (27.3)	5 (21.7)	< 0.001
Shock	3 (1.6)	0 (0.0)	4 (36.4)	11 (47.8)	< 0.001
Hypoalbuminemia (albumin < 30 g/L)	34 (18.6)	4 (23.5)	5 (45.5)	12 (52.2)	0.001
Coagulopathy	19 (10.9)	0 (0.0)	3 (27.3)	6 (30.0)	0.015
Length of hospital stay, days	23.4 (0.99)	25.5 (3.23)	24.8 (4.02)	23.1 (2.89)	0.917

Data are means (SE) and are adjusted for age and sex unless otherwise indicated percentages. Abbreviations: *COVID-19* coronavirus disease 2019, *NTF* Normal thyroid function, *NTIS* Non-thyroidal illness syndrome, *ARDS* Acute respiratory distress syndrome

The multivariate-adjusted Cox models for the primary composite endpoint including admission to the ICU, using IMV, or death events in different thyroid function categories are shown in Table 4. Compared with normal thyroid function, mild to moderate NTIS showed the highest risk for composite endpoint (HR 27.3; 95% confidence interval [CI] 7.07–105.7), severe NTIS had the second high risk for the composite endpoint (HR 23.1; 95% CI 5.75–92.8), and subclinical hypothyroidism had a slightly higher risk for the composite endpoint (HR 4.04; 95% CI 0.69–23.8), after adjusting for age, sex, smoking, systolic blood pressure, fasting glucose, using antihypertensive drugs, and using glucose-lowering drugs (Model 2). After adjustment for total cholesterol and using lipid-lowering agents (Model 3), these associations had similar changes.

**Table 4:** Hazard ratios of combined endpoint events according to different thyroid function categories among patients with COVID-19

	No. of participants	No. of cases	Person-days	Hazard ratios (95% confidence intervals)		
				Model 1	Model 2	Model 3
Combined endpoint events ^a^						
NTF	184	5	5753	1.00	1.00	1.00
Subclinical hypothyroidism	17	2	524	5.55 (1.01–30.5)	4.04 (0.69–23.8)	7.14 (1.26–40.6)
Severe NTIS	11	6	350	18.8 (5.05–69.9)	23.1 (5.75–92.8)	22.1 (6.09–79.9)
Mild to moderate NTIS	23	15	685	21.0 (6.04–72.7)	27.3 (7.07–105.7)	30.1 (9.89–91.3)

Model 1 adjusted for age and sex. Model 2 adjusted for age, sex, smoking, systolic blood pressure, fasting glucose, using antihypertensive drugs, and using glucose-lowering drug. Model 3 adjusted for total cholesterol and using lipid-lowering agents. ^a^Combined endpoint events: The primary end point was a composite of death or using invasive mechanical ventilation or admission to Intensive care unit. Abbreviations: *COVID-19* coronavirus disease 2019, *NTF* Normal thyroid function, *NTIS* Non-thyroidal illness syndrome

The multivariate-adjusted (Model 2) HRs for acute cardiac injury among patients in the normal, subclinical hypothyroidism, severe NTIS, and mild to moderate NTIS groups were, 1.00, 1.68 (95% CI 0.56–5.05), 4.68 (95% CI 1.76–12.4), and 2.63 (95% CI 1.09–6.36), respectively (Table 5). After further adjustment for total cholesterol and using lipid-lowering agents (Model 3), these associations had similar changes.

**Table 5:** Hazard ratios of acute cardiac injury according to different thyroid function categories among patients with COVID-19

	No of participants	No. of cases	Person-days	Hazard ratios (95% confidence intervals)		
				Model 1	Model 2	Model 3
Acute cardiac injury						
NTF	184	27	5753	1.00	1.00	1.00
Subclinical hypothyroidism	17	4	524	1.68 (0.58–4.86)	1.68 (0.56–5.05)	1.97 (0.67–5.78)
Severe NTIS	11	7	350	3.78 (1.57–9.14)	4.68 (1.76–12.4)	4.20 (1.78–9.90)
Mild to moderate NTIS	23	12	685	2.50 (1.13–5.52)	2.63 (1.09–6.36)	3.93 (1.96–7.89)

Model 1 adjusted for age and sex. Model 2 adjusted for age, sex, smoking, systolic blood pressure, fasting glucose, using antihypertensive drugs, and using glucose-lowering drug. Model 3 adjusted for total cholesterol and using lipid-lowering agents. Abbreviations: *COVID-19* coronavirus disease 2019, *NTF* Normal thyroid function, *NTIS* Non-thyroidal illness syndrome

The multivariate-adjusted (Model 2) HRs for acute cardiac injury among patients in the normal, low FT3 were, 1.00, 2.47 (95%CI 1.13–5.37), respectively (Table 6). After adjustment for total cholesterol and using lipid-lowering agents (Model 3), these associations had similar changes.

**Table 6:** Hazard ratios of acute cardiac injury according to different FT3 categories among patients with COVID-19

	No of participants	No. of cases	Person-days	Hazard ratios (95% confidence intervals)		
				Model 1	Model 2	Model 3
Acute cardiac injury						
Normal FT3^a^,	205	33	6396	1.00	1.00	1.00
Low FT3^b^	30	17	916	2.39 (1.20–4.77)	2.47 (1.13–5.37)	3.46 (1.90–6.31)

Model 1 adjusted for age and sex. Model 2 adjusted for age, sex, smoking, systolic blood pressure, fasting glucose, using antihypertensive drugs, and using glucose-lowering drug. Model 3 adjusted for total cholesterol and using lipid-lowering agents. ^a^Normal FT3 was defined as FT3 within 2.63–5.7 pmol/L. ^b^Low FT3 was defined as FT3 < 2.63 pmol/L. Abbreviations: *COVID-19* coronavirus disease 2019, *FT3* Free Triiodothyronine

## Discussion

COVID-19 is an entirely new disease caused by a new pathogen known as SARS-CoV-2, which had a high fatality rate during the initial phase of the outbreak. There are many challenges that arise in understanding the pathogenesis, clinical features, and prognostic factors of COVID-19. In the current study, we primarily investigated the relationship between different types of HPT axis abnormalities and the severity of prognosis in COVID-19. We found that several different types of thyroid dysfunctions were caused by COVID-19 illness, including mild to moderate NTIS, severe NTIS, and subclinical hypothyroidism. The alterations of myocardial injury indicators, and coagulation function index were observed in all three different thyroid function groups. Besides, all three different types of thyroid dysfunction were associated with the severity of prognosis of COVID-19. The mild to moderate NTIS group had the highest composite endpoint events, followed by the severe NTIS group. Compared with the NTF group, the subclinical hypothyroidism group had an increased rate of ICU admission and IMV usage. Moreover, we found that the risk of myocardial injury, was increased in both the severe NTIS and mild to moderate NTIS groups compared to the NTF group, and FT3 was a predictor of myocardial injury in these COVID-19 patients.

The present study found that the proportion of mild to moderate NTIS was 9.79% (23/235) in COVID-19 patients. The mild to moderate NTIS refers to changes in serum thyroid hormone levels observed in critically ill patients in the absence of hypothalamic-pituitary-thyroid primary dysfunction [15, 16]. Affected individuals had low T3, elevated rT3, and inappropriately normal TSH levels due to the changes in thyroid hormone binding, peripheral thyroid hormone uptake, and in the expression and activity of type 1 and type 3 deiodinase resulting from severe illness [17, 18]. Inflammatory cytokines could play a role in the deiodinase expression disorder and oxidative stress caused by the production of reactive oxygen species (ROS) and may disrupt the function of deiodinase [4]. In our study, compared with the NTF group or the subclinical hypothyroidism group, the group with mild to moderate NTIS group had higher CRP, WBC count, ESR, and procalcitonin, suggesting that NTIS may be the result of severe inflammation. In addition, troponin, CK-MB and LDH in the mild to moderate NTIS group were higher than those in the NTF and subclinical hypothyroidism groups. The proportion of D-dimer (> 0.5 mg/L) that increased in the mild to moderate NTIS group was higher than in the other three groups. Thus, patients with mild to moderate NTIS had severe inflammation, myocardial injury, and coagulation abnormalities, which might explain the highest incidence of compound endpoint in patients with mild to moderate NTIS, suggesting that mild to moderate NTIS requires special attention and may be an indicator of poor prognosis.

Approximately 4.68% (11/235) of COVID-19 patients had severe NTIS. Pathogenesis of NTIS in long-term critical illness includes suppression of hypothalamic thyrotropin-releasing hormone, accounting for persistently reduced secretion of thyroid-stimulating hormone despite low plasma thyroid hormone. In addition, existing studies have found that SARS-CoV can invade the brain tissue. In some cases distinguishing between NTIS and central hypothyroidism can be difficult, so severe NTIS may include central hypothyroidism. The global incidence of central hypothyroidism ranges from 1 in 20,000 to 1 in 80,000 in the general population [19]. The higher incidence of central hypothyroidism in our study suggested that there was a link between COVID-19 and central hypothyroidism. The exact cause of central hypothyroidism remains unknown. Specific mechanisms may include direct virus damage, hypoxia, systemic inflammation, and ischemia or microthrombus due to hypercoagulability. Despite previous studies showing low levels of ACE2 in the pituitary [20], the detection of SARS-CoV was established in the autopsied pituitary samples of a SARS patient [21]. Also, the patients with central hypothyroidism had the highest demand for oxygen support in this study, suggesting that severe hypoxia was common in patients with central hypothyroidism. Previous studies have indicated that hypoxia may cause central hypothyroidism [22]. Systemic inflammation may have an impact on pituitary function [4, 23]. Our results showed that the patients with central hypothyroidism had higher WBC count, neutrophil count, CRP and procalcitonin levels, suggesting a pronounced systemic inflammation in central hypothyroidism. Hypoxia and/or systemic inflammation can lead to hypercoagulability [24, 25].

This study found that severe NTIS had a higher proportion of D-Dimer (> 0.5 mg/L) and increased level of fibrinogen, indicating a state of hypercoagulability and even thrombogenesis in central hypothyroidism. Patients with COVID-19 may have ischemia or microthrombus in the pituitary tissue, which affects the pituitary function and leads to central hypothyroidism.

The current study found that the proportion of subclinical hypothyroidism was 7.23% (17/235) in COVID-19 patients. Subclinical hypothyroidism may be caused by the direct destruction of the thyroid by SARS-CoV-2. A previous studies [20] demonstrated an increased expression of ACE2 in the thyroid gland, suggesting that SARS-CoV-2 might access the thyroid gland through ACE2 and cause potential damages. Autopsy results of SARS patients of previous study [26] indicated that thyroid follicular cells and parafollicular cells were damaged, with a large number of follicular epithelial cells exfoliating into the follicle and undergoing apoptosis, with the follicular structure showing distortion, expansion and collapse, no significant calcitonin-positive cells were detected. Our results indicated that the prognosis of subclinical hypothyroidism was better than that of central hypothyroidism, which may suggest that an adequate HPT axis response can improve the prognosis. When the disease affects the thyroid gland, the pituitary gland reactively increases TSH synthesis or secretion to maintain normal levels of T3 and T4. However, when the pituitary reactivity is reduced due to the destruction or inhibition of the pituitary gland, and TSH cannot be compensated to maintain T3 and T4 at normal levels, central hypothyroidism ultimately occurs, accompanied by a poor prognosis [15, 27]. Therefore, the stability of the HPT axis is essential for prognosis.

Our preliminary data suggested that FT3 was a predictor of myocardial injury in COVID-19 patients. Similar to general critical illness, lower circulating T3 levels in acute myocardial infarction patients are associated with more severe clinical symptoms and poorer clinical outcomes [28]. In addition to traditional risk factors, thyroid hormone (TH) metabolic changes are associated with poor prognosis of left ventricular dysfunction in patients with acute decompensated heart failure [29, 30]. TH receptors exist in myocardial and vascular tissues, and small changes in TH concentration can affect cardiovascular physiology [31]. Thyroid dysfunction is a potential mechanism for cardiovascular disease, including endothelial dysfunction, changes in blood pressure, myocardial systolic and diastolic dysfunction, and dyslipidemia [31]. Our results showed that the proportion of acute cardiac injury in COVID-19 patients with severe NTIS and mild to moderate NTIS significantly increased, and subclinical hypothyroidism had the highest levels of blood lipids among the four groups. Thus, this study suggested that changes in thyroid hormone levels due to the influence of the HPT axis may be a predictor of myocardial injury.

Critically ill patients with COVID-19 usually require corticosteroids therapy which is known to cause inhibition of TSH secretion [32]. We excluded patients requiring long-term corticosteroids therapy. Although thyroid function was measured in our enrolled patients on the first or second day of admission, 4.3% (10/235) patients were treated with corticosteroids before thyroid function measurement. We performed statistical analysis for including or excluding them. The patients’ exclusion from the analysis did not contribute to a significant change in the results, and thus we chose to retain them in the study.

The current study has certain limitations. First, most of the COVID-19 patients did not undergo a dynamic review of thyroid function, and the dynamic changes of the HPT axis were not shown. Second, reverse triiodothyronine were not measured, therefore partial central hypothyroidism and NTIS were difficult to distinguish. Third, the study sample was not large, and the effect of the intervention on prognosis was not designed in advance, so caution must be attached while interpreting some of the results. Fourth, it is not yet possible to distinguish in which proportions the virus is directly responsible for the dysfunction and in what proportion a subclinical alteration not previously diagnosed can make it susceptible to infection.

## Conclusions

In conclusion, our study showed that the suppression of the HPT axis could be a common complication in COVID-19 patients and an indicator of the severity of prognosis. Compared with the NTF, patients with mild to moderate NTIS and severe NTIS had a worse prognosis and increased risk of myocardial injury. Our study suggests that mild to moderate NTIS and severe NTIS could be associated with the severity of prognosis of COVID-19.

## Data Availability

The datasets generated during and analyzed during the current study are available from the corresponding author on reasonable request.

## Abbreviations

ARDS: acute respiratory distress syndrome
ACE2: angiotensin-converting enzyme 2
COVID-19: coronavirus disease 2019
CI: confidence interval
COPD: chronic obstructive pulmonary disease
CRP: c-reactive protein
ESR: erythrocyte sedimentation rate
FT3: Free Triiodothyronine
FT4: Free Thyroxine
HRs: hazard ratios
HPT: hypothalamic-pituitary-thyroid
ICU: intensive care unit
IMV: invasive mechanical ventilation
NTF: normal thyroid function
NTIS: non-thyroidal illness syndrome
SARS-CoV-2: severe acute respiratory syndrome coronavirus 2
TH: thyroid hormone
TSH: Thyroid-stimulating hormone
WHO: World Health Organization
